# Supplementary material for: In Vivo and In Vitro Evidence for an Interplay between the Glucocorticoid Receptor and the Vitamin D Receptor Signaling
Source: Cells. 2023 Sep 15;12(18):2291. doi: 10.3390/cells12182291 (PMC10527904; doi:10.3390/cells12182291)
Supplement: Supplementary file 1 [file cells-12-02291-s001.zip › cells-2454331-supplementary final 2.pdf]

Article

In vivo and *in vitro* evidence for an interplay between the glucocorticoid receptor and the vitamin D receptor signaling

Maud Bagnoud[1], Jana Remlinger[1], Marine Massy[1,2], Dmitri Lodygin[3], Anke Salmen[1], Andrew Chan[1], Fred Lühder[3], Robert Hoepner[1]

[1] Department of Neurology, Inselspital, Bern University Hospital and Department of Biomedical Research, University of Bern, Bern, Switzerland
[2] Graduate School for Cellular and Biomedical Sciences, University of Bern, Bern, Switzerland
[3] University Medical Center Göttingen, Institute for Neuroimmunology and Multiple Sclerosis Research, Göttingen, Germany
* Correspondence: maud.bagnoud@insel.ch; Tel.: +41 31 632 86 36

Figure S1: Nuclear translocation of the GR *in vitro*

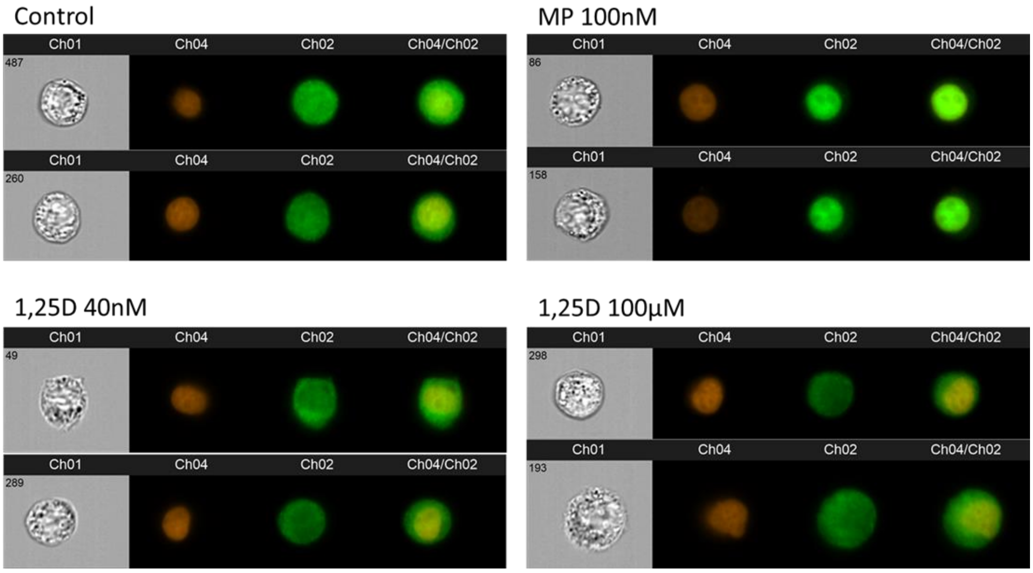

Figure S1: Hela ω3 cells expressing mCherry-H2B in the nucleus were incubated for 2h with control, MP 100nM, or different concentrations of calcitriol (40nM, 400nM, 4µM, 100µM). Channel 4 (Ch04) shows the nucleus, channel 2 (Ch02) the GR, and Ch04/Ch02 the overlay. ImageStream system. Abbreviations: MP: methylprednisolone, 1,25D: calcitriol.

Figure S2: Calcitriol influences VDR and GR protein expression and induces their nuclear co-localization *in vitro*

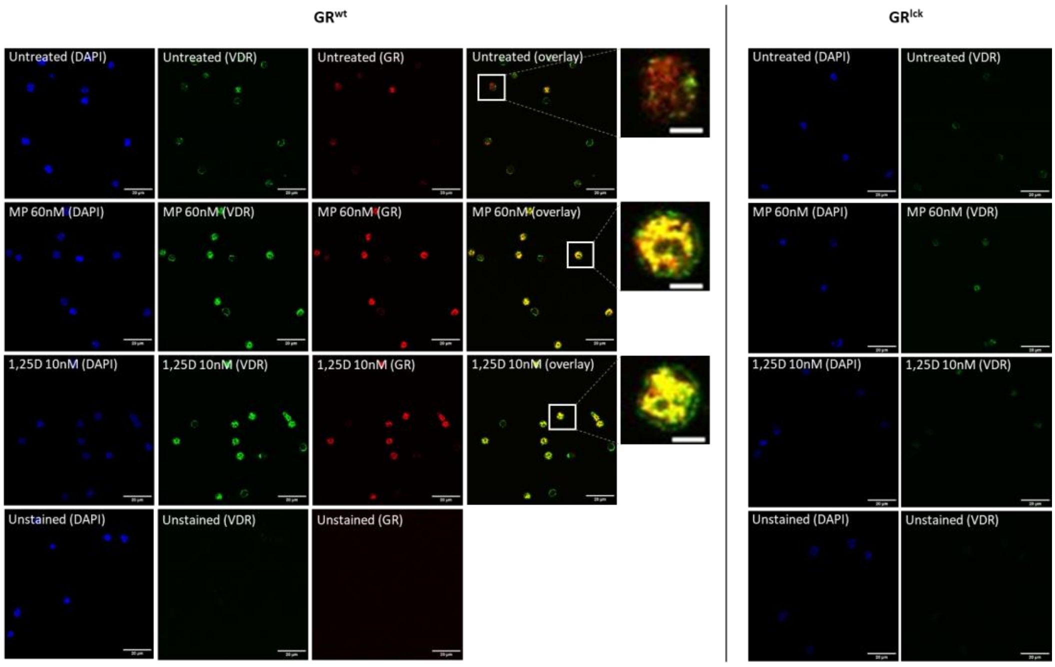

Figure S2: VDR and GR protein expression and their co-localization were analyzed in stimulated splenic-derived CD3+ T cells of WT and GR^{lck} and in WT mice, respectively, treated with control, MP 60nM or calcitriol 10nM (stimulus conA 1.5µg/ml; 2h). DAPI is shown in blue, the vitamin D receptor in green (AF 488), and the glucocorticoid receptor in red (AF 555). The bar scale in the images showing the magnification of one cell represents 3µm whereas in the other images it represents 20µm. Confocal microscopy. Abbreviations: DAPI: 4′,6-diamidino-2-phenylindole, MP: methylprednisolone, 1,25D: calcitriol.
